# Lipid Screening, Action, and Follow-up in Children and Adolescents

**DOI:** 10.1007/s11886-018-1014-7

**Published:** 2018-08-09

**Authors:** Albert Wiegman

**Affiliations:** Department of Pediatrics, Amsterdam UMC, University of Amsterdam, Amsterdam, The Netherlands

**Keywords:** Adolescent, Child, Familial hypercholesterolemia, Lipids, Genetic screening

## Abstract

**Purpose of Review:**

To create awareness for the devastating influence of high cholesterol in familial hypercholesterolaemia (FH) on vessel walls. Persons with high LDL-C and a known mutation associated with FH have a 22-fold increase in CVD compared with those with a normal LDL-C and no genetic mutation. If the awareness of the need to diagnose and treat this genetic disorder at an early stage increases, great atherosclerotic impact later in life could be avoided. Every minute a child with heterozygous FH is born somewhere in the world and every day a child with homozygous FH is born.

**Recent Findings:**

Recent findings include effective therapy on statins from the age of 6 years, with already normalization of the intima-media thickness within 2 years. Newer types of drugs, with the same safety profile and perhaps even more effective, will become available in childhood in the near future. Open for discussion will be whom to treat and with what type of treatment. Next generation sequencing will perhaps easily select those in need of treatment and those at risk of adverse effects.

**Summary:**

At the end of this review, statements and recommendations for children and adolescents with heterozygous FH are listed.

## Introduction

There is a lot one can do about lowering cholesterol, especially in children. The question is whether high cholesterol is that dangerous in childhood. To be honest, we do not know for sure, but we do know it depends on background. In this paper, I will focus on familial hypercholesterolemia (FH), an autosomal dominant inherited disorder that predisposes to early onset of atherosclerosis. A disorder in which the answer definitely is a Yes. In The Netherlands, cascade screening for FH has been on-going since 1994, funded by the Ministry of Health. Owing to that program, many children with this heritable disorder were traced and referred to our pediatric lipid clinic because of the great danger later in life. Unfortunately, after 20 very successful years, the funding was halted in 2014, on the presumption that three quarters of all people with FH were detected, assuming a frequency of 1 in 400. In reality, not even half of all Dutch persons suffering from this disorder were discovered; the same applied for the children. The estimated prevalence of heterozygous FH (heFH) in the Netherlands turned out to be 1:250 [1, 2], and the prevalence of homozygous FH (hoFH) 1:250.000 (= 1/250 * 1/250 * 1/4). Other investigators in Denmark, USA, UK, and West Australia confirm this prevalence, making it not exclusively limited to the Dutch borders and thus one of the most common single gene disorders in the world. [3, 4••, 5••, 6] Nowadays, a group of Dutch hospitals, in collaboration with general practitioners, try to pursue this productive screening program.

The underlying defect is, in almost 90% of the molecular diagnosed FH patients, a mutated low-density lipoprotein receptor (LDLR), which results in a more than doubling of the plasma low-density lipoprotein cholesterol (LDL-C) levels. In about 10%, a mutation in the gene-encoding apolipoprotein B, which is the major protein of the LDL particle, is the cause. In less than 1% a mutation in the gene-encoding proprotein convertase subtilisin/kexin type 9 (PCSK9) is identified, involved in degrading the LDLR. Even in childhood, with a mutation detection rate of more than 90% [7, 8], we still lack 10% in highly probable FH phenotypes.

Primates and even sharks share the same amino acid sequencing of the LDLR. So, this receptor must have been assembled already more than 450,000,000 years ago, before sharks and primates took separate evolutionary paths. Time enough for this gene to collect mutations. Worldwide over 1300 different pathogenic variations have been identified. This molecular heterogeneity is because FH is not a fatal disease at a young age. Carriers of a mutation get many chances to transmit to their offspring. Of course, this not only happens in The Netherlands.

## Homozygous FH

**Fig. 1:**
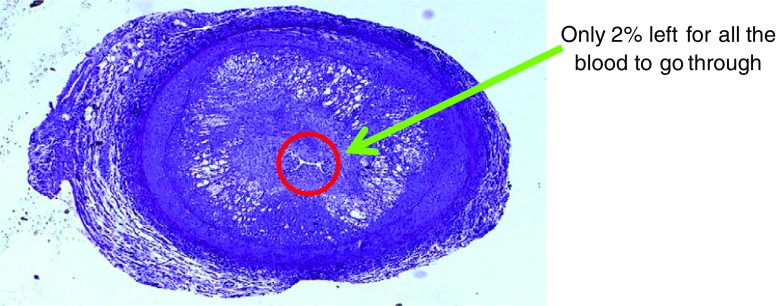
Boy with homozygous FH died suddenly at age of 4; at autopsy, 98% occlusion of left coronary artery. (Reprinted from: Widhalm K, et al. J Pediatr 2011;158:167, with permission from Elsevier) [9]

**Fig. 2:**
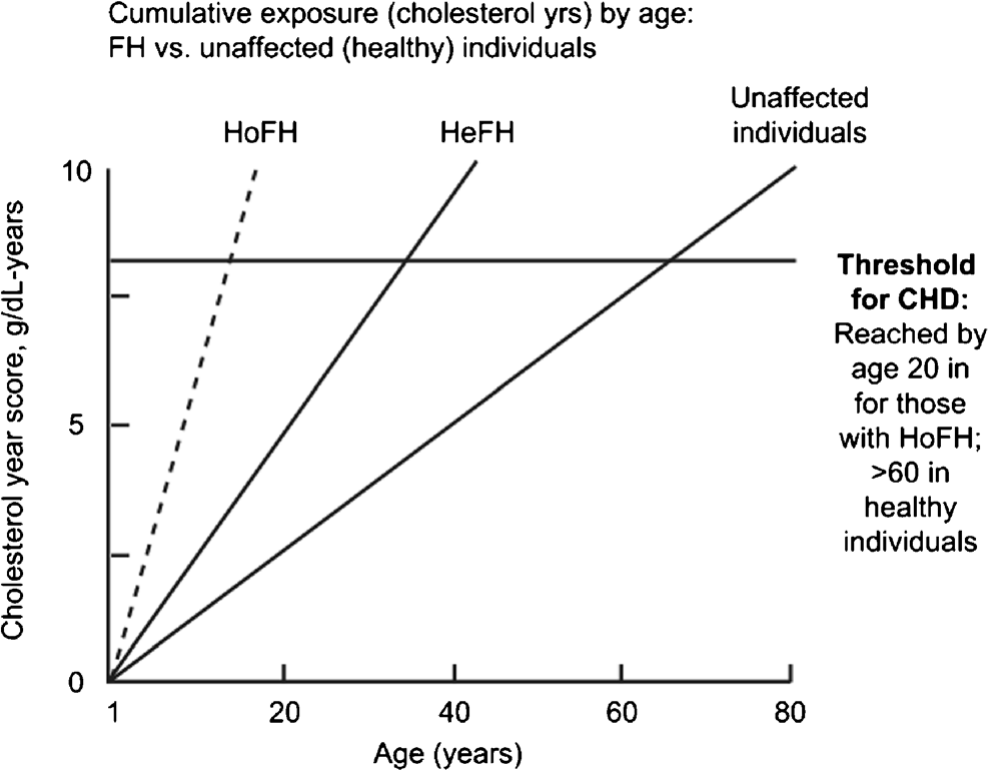
Cumulative low-density lipoprotein (LDL) exposure (expressed as grams of cholesterol per year) over a lifetime in familial hypercholesterolemia patients and normal individuals. CHD, coronary heart disease; FH, familial hypercholesterolemia; HeFH, heterozygous familial hypercholesterolemia; HoFH, homozygous hypercholesterolemia. (Reprinted from: Raal FJ, Santos RD. Atherosclerosis. 2012 Aug;223(2):262-8, with permission from Elsevier) [11]

Although hoFH is rare, an emphasis on this disorder and early recognition are extremely important because it may rapidly lead to juvenile onset of cardiovascular disease (CVD) and premature death. Between 2010 and 2013, four children died from ischaemic heart disease at the age of 3.5, 4, 4.5, and 5 years old in Germany, Austria, Switzerland, and Italy, respectively, even though they were already diagnosed at the age of 1 and 2 [9, 10, and personal communication] (Fig. 1). From that moment on, every center with hoFH children became aware of the necessity to start treatment as early as possible. So, to an even greater degree than heFH, hoFH can be disabling at a young age and shorten life expectancy (Fig. 2). If an untreated LDL-C exceeds 13 mmol/L (500 mg/L) and pediatric manifestations include CVD and/or xanthomas, homozygosity is assumed.

## Physical Characteristics

In childhood, typical physical characteristics of FH, like arcus cornealis, xanthelasmata and xanthomas, especially in the Achilles tendons, in the extensor tendon on the dorsum of the hand and interdigital, are rare. If one or more are present, it is almost pathognomonic for FH. In our center, more than half of 28 children with hoFH have xanthomas present before the age of 10 years. Whereas only 58 out of 2238 children with heFH proven by a pathogenic mutation have virtual diagnostics, not even 3% of the heterozygous children who were referred to our center. Much more present is pain in the Achilles tendon: in an unpublished substudy in 529 children with heFH, 30% suffered from painful Achilles tendons, whereas none of their unaffected siblings did.

## Screening Strategies

Different screening strategies are currently recommended to identify children with FH to initiate early lipid management. Screening can be done via cholesterol testing, genetic analysis, or both. However, these strategies are characterized to date by low adherence by the medical community and limited compliance by parents and children [12]. Cascade screening can reduce the average age at which individuals with FH are diagnosed and increase the percentage of individuals receiving lipid lowering therapies, potentially resulting in reductions in LDL-C and CVD. Cascade screening in our country identified on average eight relatives with FH for each index case and significantly increased the proportion of FH patients receiving treatment [13]. As a co-product, another nine relatives received the comforting message not to be affected.

## Pedigrees

**Fig. 3:**
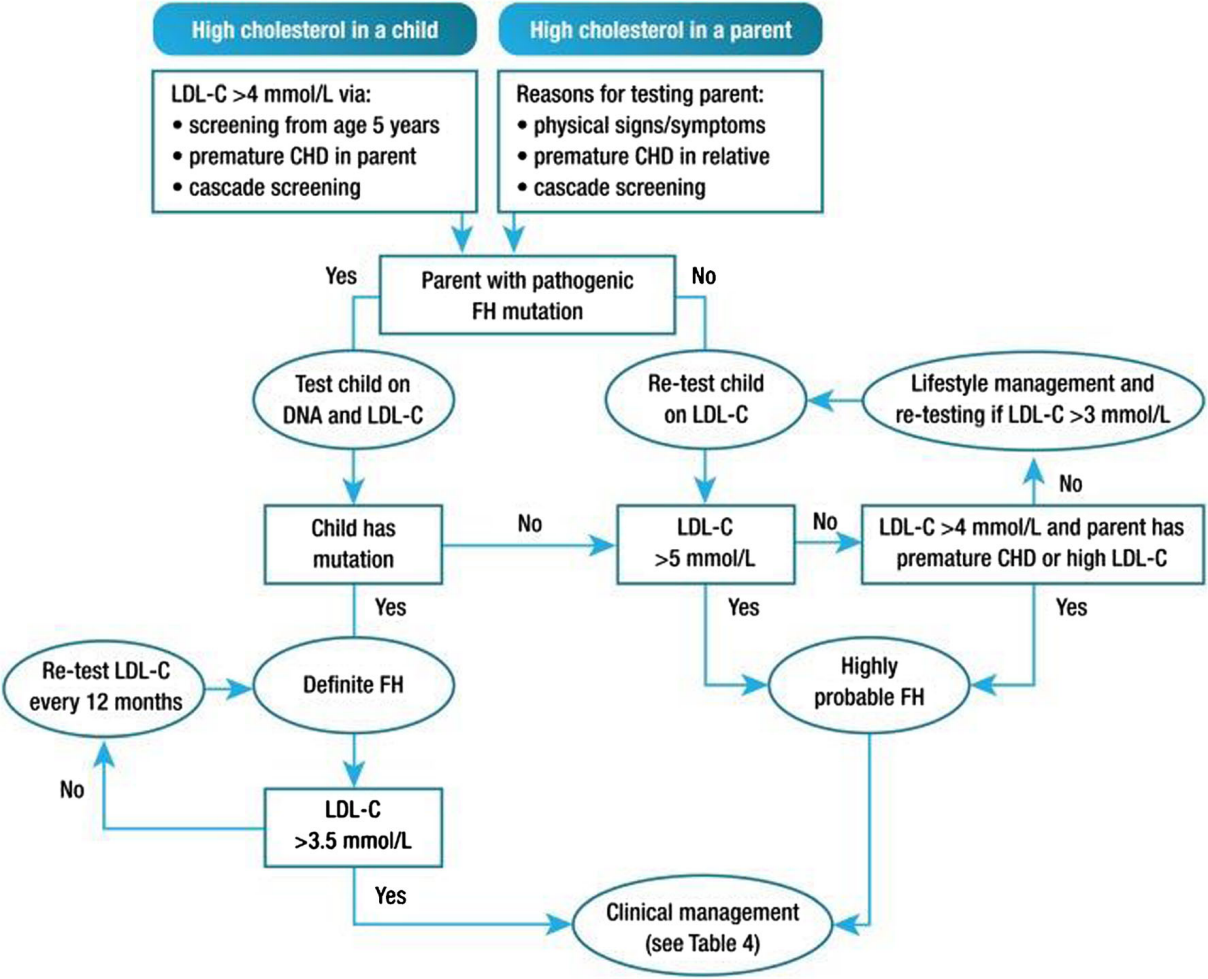
Potential strategy for diagnosis of familial hypercholesterolaemia in children and adolescents. CHD, coronary heart disease; FH, familial hypercholesterolaemia; LDL-C, low-density lipoprotein cholesterol. (From: Wiegman A, et al. Eur Heart J 2015;36:2425-37, by permission of Oxford University Press) [14]

Cascade screening starts with drawing a pedigree. One will be confronted with premature CVD running in the family (men <55 years of age and women <65 years of age), most of the time without skipping generations. However, premature CVD as a factor in all risk models will vanish with early adequate treatment, re-increasing the risk for next generations. Recently, the European Atherosclerosis Society (EAS) proposed heFH diagnostic criteria for children based on LDL-C cut-off values and family history of hypercholesterolemia and/or premature CVD [14] (Fig. 3).

When in a post hoc analysis different diagnostic criteria were applied to children with clinically diagnosed FH, those proposed by EAS showed a reasonable balance between sensitivity and specificity in the identification of carriers of LDLR mutations. In particular when children with LDL-C >190 mg/dL were included in the genetic analysis, the detection of mutant LDLR alleles reached 70%. Both Simone Broom Register Group and Dutch Lipid Network Criteria overall showed a poorer performance [15].

## Risk Factors

In this manuscript, we will focus on FH. However, atherosclerosis and its clinical consequence are multifactorial in origin. Therefore, it is important, especially in patients with FH, to pay sufficient attention to all the other risk factors that constitute their total CVD risk. In children and adolescents, it is essential to abstain from tobacco smoking, to exercise regularly, and to keep up with a well-balanced and healthy diet, the primordial and primary prevention of CVD. Every clinical visit, optimally before puberty starts, smoking habits, exercise and diet, as cornerstones of a normal lifestyle should be discussed. Hypertension, diabetes, and obesity are classic modifiable risk factors, although in children with molecular proven FH relatively rare.

## Need for Early Therapy

Lifelong elevation of cholesterol levels on a genetic basis imposes substantially greater risk than acquired hyperlipidemia in midlife. As shown recently by Khera et al, compared with persons with a normal LDL-C and no genetic mutation associated with FH, those with high LDL-C (>5 mmol/L) and no mutation have a 6-fold increase in CVD, whereas those with high LDL-C and a known mutation have a 22-fold higher risk [4••]. In 2008, outcomes from Neil et al [16] already confirmed the importance of early identification and treatment of affected heterozygous individuals, since the major benefit of statin treatment appears to be in the primary prevention of fatal CVD. With earlier diagnosis, it should be possible to prevent any excess coronary mortality in early adulthood. It supports a strategy of cascade testing to identify the affected relatives and probands. Since LDL-C levels are elevated from birth onwards, testing must include children, who as yet have the highest rates of underdiagnosis [17].

## Carotid Intima Media Thickness (cIMT)

Population studies have shown that cIMT has been consistently associated with future atherosclerotic disease. In children with heFH increased cIMT is already manifested [18, 19•]. Reduction in cIMT progression in children with heFH might reduce the risk of future CVD.

## Nonpharmacologic Treatment

A recent systematic review about dietary interventions in children and adults with FH stated that no conclusions can be made about the effectiveness of a cholesterol-lowering diet or any of the other dietary interventions suggested for FH on the prevention of CVD [20]. Currently, the use of foods supplemented with plant sterols or stanols is only recommended for children from the age of 5 years [21].

## Statins

Statins are the cornerstone of FH treatment and they appear to be safe and effective in children. However, most studies done in children had a short-term follow-up period. Recently, a study on statins with 10 years of follow-up has been published, the longest follow-up study thus far. This showed that long-term statin treatment initiated in childhood was associated with normalization of cIMT progression during aging. In terms of long-term safety, this study did not reveal a significant difference in laboratory parameters, and overall statin therapy was well tolerated [22••]. In childhood, statins are safe. Three systematic reviews did not find any difference between the statin group and control group in the proportion of participants who experienced a clinically significant increase in liver transaminase values (over 3-fold increase in alanine transferase or aspartate aminotransferase) or creatine kinase values (over 10-fold increase); or with respect to their growth and sexual maturation measured by the Tanner staging; or on academic performance. Overall, the data suggest the risk of adverse events in children treated with statins is similar to that observed in statin-treated adults over the short term and the adverse event rate was the same between statin group and placebo group. These findings are similar in all reported systematic reviews [23–25]. In our cohort, more than 97% of all children on statins are responding extremely well. For those who develop a mild to moderate intolerance to statins, another statin may be tried before progressing to the evidence-based non-statin therapies. In case of heFH, statins are available from the age of 8 years (in near future from age 6 years), in case of hoFH as early as diagnosed.

## Rosuvastatin and cIMT Reduction

**Fig. 4:**
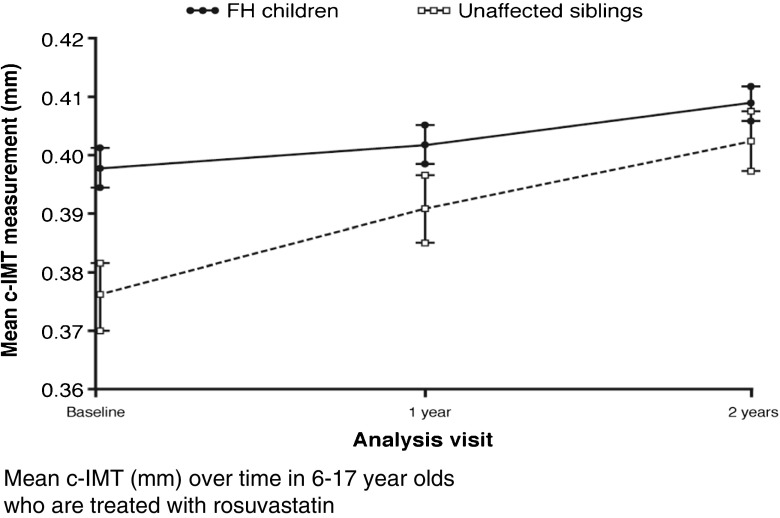
Effect of rosuvastatin on carotid intima-media thickness in children with heterozygous familial hypercholesterolemia. (From: Braamskamp MJAM, et al. Circulation 2017;136:359-66, with permission) [26••]

Recently we published an important outcome of a study to assess the efficacy of rosuvastatin in children from 6 till 17 with heFH. Their cIMT was significantly greater at baseline compared with their unaffected siblings. Rosuvastatin treatment for 2 years resulted in significantly less progression of increased cIMT in heFH children than in untreated unaffected siblings. As a result, no difference in cIMT could be detected between the two groups after 2 years of rosuvastatin. These findings support the value of early initiation of statin treatment for LDL-C reduction in children with heFH (Fig. 4) [26••].

## Intensive Treatment in hoFH Children

In children with hoFH, to prevent or delay CVD, early and aggressive cholesterol-lowering treatment is warranted [27]. Starting with a statin, with or without ezetimibe, as soon as the diagnosis is clear, delays cardiovascular events and prolongs survival as shown in a retrospective cohort study [28]. Currently, we published the first-ever pediatric hoFH statin trial, demonstrating safe and effective LDL-C reduction with rosuvastatin 20 mg alone or added to ezetimibe and/or apheresis [29]. In case there is not enough decrease in LDL-C levels on lipid-lowering drugs, an LDL-apheresis is the treatment of choice and can improve CVD outcome [30]. A single treatment can decrease plasma LDL-C by 55%–70 % relative to pretreatment levels [27]. This reduction, however, is temporary, and treatment once per 1 or 2 weeks is therefore desirable [31, 32]. When performed once a week, mean LDL-C levels can come to target levels [27]. Data on LDL apheresis in children is limited, but several case series and case reports show LDL apheresis to be a safe and effective treatment for hoFH in children [33–35]. Current guidelines recommend starting apheresis in those who need this as soon as possible, ideally by age 5 years, but no later than age 8 years [27].

## Lipoprotein (a)

High lipoprotein (a) [Lp(a)] is a risk factor for CVD, independent from FH. Lp(a) is a liver-synthesized LDL-like particle containing a glycoprotein, apo(a), which is covalently bound to Apo B. High plasma Lp(a) levels are associated with coronary artery disease, peripheral artery disease, and stroke [36, 37]. Although the role of plasma Lp(a) levels as risk factor for CVD in heFH has been controversial, some retrospective and cross-sectional studies have shown that Lp(a) is indeed an independent risk factor for CVD in these patients [38, 39]. In case of premature CVD in close relatives, the influence of high Lp(a) should be taken into consideration, although in children this needs further investigation.

## Statin Myopathy and SLCO1B1 Mutation

It is interesting to find out why statin myopathy is so rare in children with molecular proven FH. But it is of even greater interest to know the mechanism and role of statin-transporters in the few children who do suffer from statin myopathy. SLCO1B1 521C is currently the only clinically relevant pharmacogenetics test regarding statin toxicity [40]. This polymorphism in SLCO1B1 results in decreased transport of statins into the hepatocytes and significantly affects the risk of statin toxicity, especially in simvastatin treatment. SLCO1B1 521C homozygous carriers were approximately three times more likely to be statin intolerant compared with wild type. Heterozygote and homozygote carriers were 4.5 and 16.9 times more likely to develop statin myopathy, respectively, compared with wild type [41]. Many other statin transporters should be investigated for their potential role. With next generation sequencing, those at risk for statin myopathy can be indicated, and prescribing specialists are warned.

## Anti-PCSK9

A new type of drug, the proprotein convertase subtilisin/kexin type 9 (PCSK9) inhibitor, can markedly decrease LDL-C levels and claims to have cardiovascular benefits. The addition of ezetimibe to statin therapy may decrease LDL-C by about another 15% to 20%, and PCSK9 inhibitors such as evolocumab or alirocumab can drive LDL-C levels to 30 mg/dL or even lower [42]. Thus far, only one trial with anti-PCSK9 treatment included adolescents from the age of 12 years. In young patients with hoFH not receiving apheresis, monthly evolocumab 420 mg administered with stable background statin therapy with or without ezetimibe significantly reduced LDL-C levels by 31%. In patients in whom at least one mutation in the LDLR was associated with defective activity, the LDL-C reduction was 41%. Evolocumab was well tolerated and offers an effective additional option to reduce LDL-C as part of the clinical management of these patients [43]. In children with heFH, this new type of drug should be earmarked for those who are statin intolerant or lack achieving target goals with statins alone or even in combination with ezetimibe. Further investigation should be carried out to verify the safety and efficacy of anti-PCSK9 in this age group.

## Conclusion

Early detection and treatment of FH in childhood or adolescence will gain decades of healthy life. This is because FH induces lifelong increased risk of atherosclerosis beginning in childhood, causing premature CVD. There is considerable data available indicating that lipid-lowering statin therapy in children with heFH shows efficacy in reducing LDL-C and inhibiting atherosclerosis progression, with a good safety profile and no influence on growth and maturation. Therefore, early detection and treatment of the disease in childhood is highly justified. Although not always feasible in a clinical setting, family cascade screening is undoubtedly the most cost-effective strategy for detecting FH at early ages. New types of drugs should be further investigated and restricted for those at highest risk of premature CVD or statin myopathy.

Statements/recommendations for children and adolescents with heFH:

- HeFH can be highly disabling at a young age, devastating decades of life
- Lifestyle, especially non-smoking, must be incorporated before puberty, to adhere more easily
- Therefore, early detection of heFH is needed
- In case of heFH, statin treatment is available from the age of 8 years (in the near future from age 6 years), in case of hoFH as early as diagnosed
- Long-term safety is important, not only for direct health, but for lifelong adherence
- Girls should start treatment as early as boys, so they can safely stop during pregnancy
- Signs and symptoms in heFH are rare, except pain in Achilles tendon (30%)
- DNA testing is of great help,
  - not only for adherence to drugs, but
  - for awareness that atherosclerosis starts at birth, and
  - for heFH to be given on to the next generation
- Premature CVD as a factor in all risk models will vanish with early adequate treatment, giving extra risk for the next generation
- Drug treatment is safe and tolerable at young age
- Treatment with stronger statins is sometimes needed in or even before puberty
- For the rest of life, LDL-C should be normal, that is in between 1.7 (65) and 3.4 (130) mmol/L (mg/dL)
- In case optimal LDL-C goals could not be achieved, ezetimibe is the first additive, and if incapable, in rare cases anti-PCSK9 is an alternative
- In case of statin intolerance, research should be done to prove anti-PCSK9 therapy of value
